# Effect of lubricants on the rotational transmission between solid-state gears

**DOI:** 10.3762/bjnano.13.3

**Published:** 2022-01-05

**Authors:** Huang-Hsiang Lin, Jonathan Heinze, Alexander Croy, Rafael Gutiérrez, Gianaurelio Cuniberti

**Affiliations:** 1 Institute for Materials Science and Max Bergmann Center of Biomaterials, TU Dresden, Dresden, Germany

**Keywords:** lubricants, MD simulation, rotational transmission, solid-state gears

## Abstract

Lubricants are widely used in macroscopic mechanical systems to reduce friction and wear. However, on the microscopic scale, it is not clear to what extent lubricants are beneficial. Therefore, in this study, we consider two diamond solid-state gears at the nanoscale immersed in different lubricant molecules and perform classical MD simulations to investigate the rotational transmission of motion. We find that lubricants can help to synchronize the rotational transmission between gears regardless of the molecular species and the center-of-mass distance. Moreover, the influence of the angular velocity of the driving gear is investigated and shown to be related to the bond formation process between gears.

## Introduction

In mechanical systems, lubrication is the most common way to reduce friction and wear [1–4]. The idea of lubricants is preventing direct contact between surfaces to avoid dry friction from asperities and wear. Hence, the desirable lubrication regime would be hydrodynamic or elastohydrodynamic lubrication in the Stribeck curve [5]. The former corresponds to the situation that surfaces are completely separated by a fluid. The latter is similar but surface deformations are taken into account due to high pressure at intermediate sliding velocities. On the macroscopic scale, the hydrodynamics of the fluid can be analyzed by computational fluid dynamics (CFD) [6–7], which is based on solving the Navier–Stokes equation [8–9] or Reynold equation [10] for the thin-film fluid. One obtains several fluid properties such as pressure, velocity, shear stress, density and strain rate. In the case of the gear–oil–gear system, several studies based on the CFD simulation have been reported [11–18]. However, most of the simulations for this type of problem are carried out with fixed rotational speed for both gears. In this case, the gears will never be in contact with each other and only lubricant properties are calculated accordingly by the dynamical meshing at each time step. Moreover, as the system dimension approaches the nanoscale, the situation becomes very different since a continuum description of the materials might not be sufficient.

The development of the atomic force microscope (AFM) [19] and the scanning tunneling microscope (STM) [20–21] has allowed for visualization and manipulation of nanoscale gears [22]. Those gears can be either solid-state gears or molecular gears, which are created by top-down approaches (e.g., using focused ion beams [23] or electron beams [24–25] to etch the substrate) or bottom-up approaches such as chemical synthesis [26–27]. The ultimate goal for those miniaturized gears is to implement nanoscale mechanical systems such as nanorobots [28] or mechanical calculators such as the Pascaline [29]. This draws a lot of attention to issues such as triggering rotations on a surface [30–40], collective rotations [41–48] and rotational dissipation [49]. To proceed further, one may ask if lubricants can provide the same functionality as in the macroscopic case and are able to improve the transmission efficiency.

Consider the case where the lubricant film within the contact area consists only of a small number of molecules. In this case, the pressure and velocity distribution are not well defined and one has to resort to an atomistic description, for example, via molecular dynamics (MD) simulations. Also, the contact mechanics at the nanoscale is very different from the macroscopic case since specific pair interactions have to be taken into account by, for example, Lennard-Jones potentials [50]. Several works based on MD simulations were performed to study the shear viscosity in either bulk lubricants [51–53] or lubricants confined by two surfaces [54–55]. However, to date, MD simulations for the gear–lubricant–gear case are still missing. A deeper understanding of how different lubricants interact with gears during rotational transmission is hence highly desirable [56].

The paper is organized as follows: In the Methodology section, we introduce the setup of the gear–lubricant–gear system and details about the MD simulations. In Results and Discussion, we investigate the rotational transmission between diamond-based solid-state gears immersed in different lubricant molecules and with various center-of-mass distances. This is followed by a study of the angular velocity and the relation to the bond formation between gears.

## Methodology

In this section, we introduce how the system is defined and we specify the simulation protocols used in the MD simulations.

### Setup

**Figure 1:**
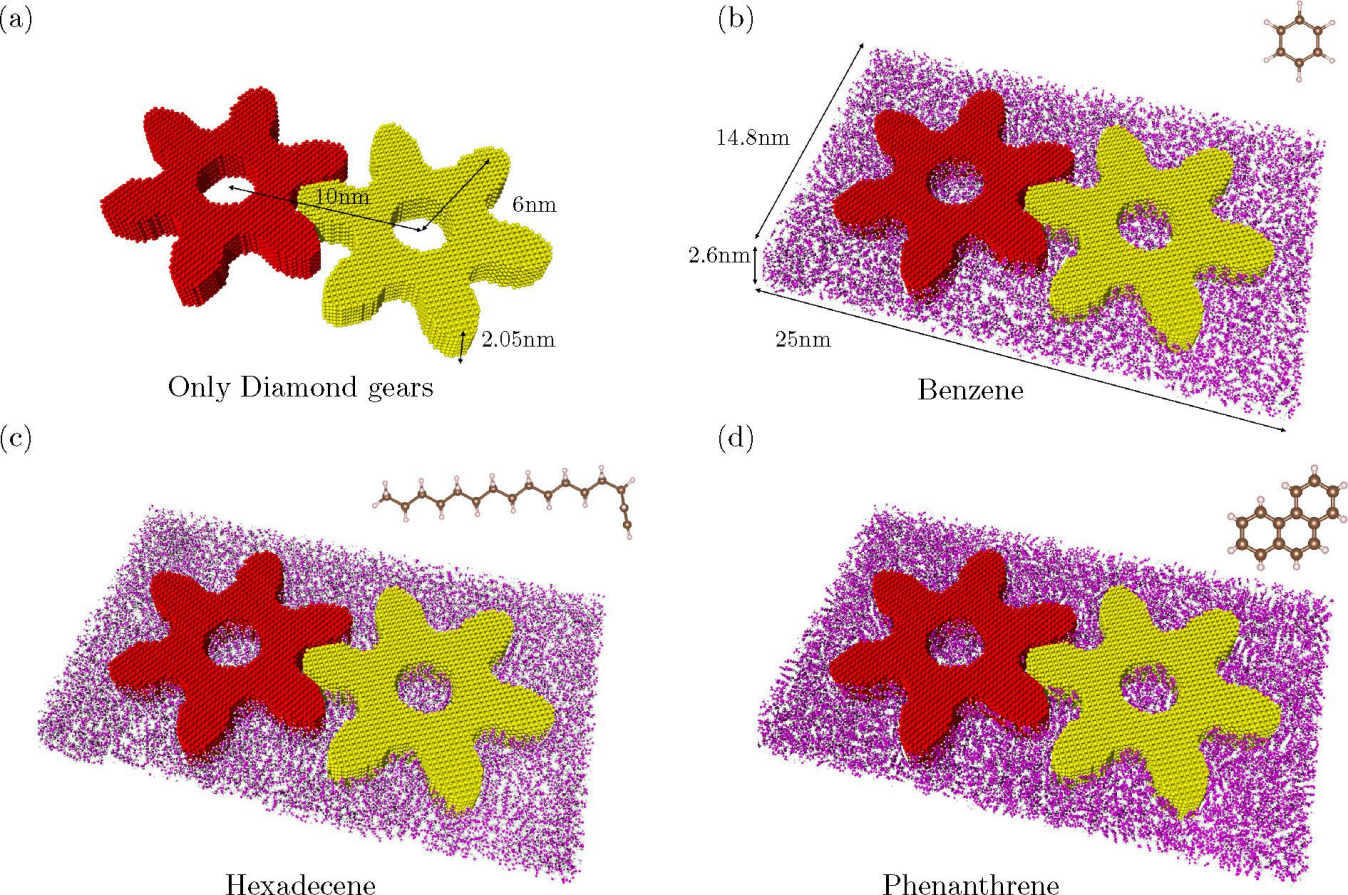
A schematic illustration of two interlocked diamond involute solid-state gears (red and yellow) with separation distance 10 nm, tip radius 6 nm and thickness 2.05 nm; (a) without lubricants and lubricated by (b) benzene, (c) hexadecene and (d) phenanthrene molecules where the purple and white atoms denote the carbon and hydrogen atoms of the lubricant, respectively. The dimension of the lubricant layer shown here is 25 × 14.8 × 2.6 nm^3^ for better visibility, but note that the actual thickness of the lubricant layer is 5.0–5.5 nm to ensure that the gears are fully immersed.

To start our study of how different lubricants can affect the rotational transmission at the nanoscale, we consider the system shown in Figure 1. First, we design two diamond solid-state gears with thickness 2.05 nm, a circular hole in the middle (with radius *r* = 1.5 nm) and six involute teeth, which are optimized for transmission in classical rigid-body gears, with tip radius *r*_tip_ = 6 nm. The center-of-mass distance is chosen to be 10 nm, which is large enough to ensure that there are still some lubricant molecules between the gears during rotation. To confine the gear rotation, we define an artificial Lennard-Jones (LJ) plane with parameters given below and an initial distance of 9.3 Å below the gears with periodic boundary conditions in both *x*- and *y*-directions. We choose this artificial LJ plane instead of a substrate with a specific arrangement of atoms to reduce the computational cost and to focus on the rotational transmission.

Next, we use GROMACS [57] to prepare the system such that the two gears are immersed in the lubricant. We have chosen three different lubricants: benzene, hexadecene and phenanthrene as shown in Figure 1b, c and d, respectively. Those lubricants are chosen due to their structural simplicity and because of being liquid at room temperature. Note that in Figure 1 we denote the thickness of the lubricated layer as 2.6 nm, which is only for better visibility since the actual thickness of the lubricant layer is 5.0–5.5 nm to ensure that the gears are immersed. Finally, the whole system is optimized by using the conjugate gradient method implemented within LAMMPS [58].

### Molecular dynamics

In this study, we use LAMMPS to perform the MD simulations. For the force fields, we choose the adaptive intermolecular reactive empirical bond order (AIREBO) potential [59]. This potential was designed for hydrocarbon systems and can reach reasonable densities for the molecules we will use later. We have used two different protocols. For protocol A, we use (i) AIREBO for carbon interactions within the gears, (ii) AIREBO for gear–lubricant interactions and (iii) a 12-6 Lennard-Jones potential with ε = 2.875 meV, σ = 3.5 Å [60] and cutoff distance 3σ for gear–gear interactions. Note, that the LJ parameters are chosen differently to the ones in AIREBO in order to mimic hydrogen passivation. This protocol is only used to study the transmission between gears, since no bond formation will happen between gears.

For protocol B, we use AIREBO for all interactions. In this case, we allow for bonds to be formed between gears, since the AIREBO potential is reactive. One might wonder how gear surface passivation (e.g., atoms saturated by hydrogen) can affect the bond formation in this case. For perfect passivation, we should not expect any bond formation under normal conditions. In reality, the gear surface passivation should be somewhere between no passivation and complete passivation. Therefore, in our case, it can be viewed as the easiest case to have bond formation. Also, to constrain the rotational axle, we connect a stiff spring with spring constant *k* = 1600 N/m (1000 eV/Å^2^) to the center-of-mass of either gear. To fix the temperature, the lubricant molecules are subject to the canonical ensemble (NVT) implemented by the Nosé–Hoover thermostat [61–62] at *T* = 400 K (such that all lubricants are in liquid phase) and the gears are subject to the microcanonical ensemble (NVE) in order to explicitly monitor the energy transfer during our simulation.

## Results and Discussion

In this section, we present the results for the rotational transmission with and without lubricant and discuss the effect of the center-of-mass distance and of the angular velocity. Finally, we look into the bond formation behavior between gears.

### Lubricants and distance dependence

**Figure 2:**
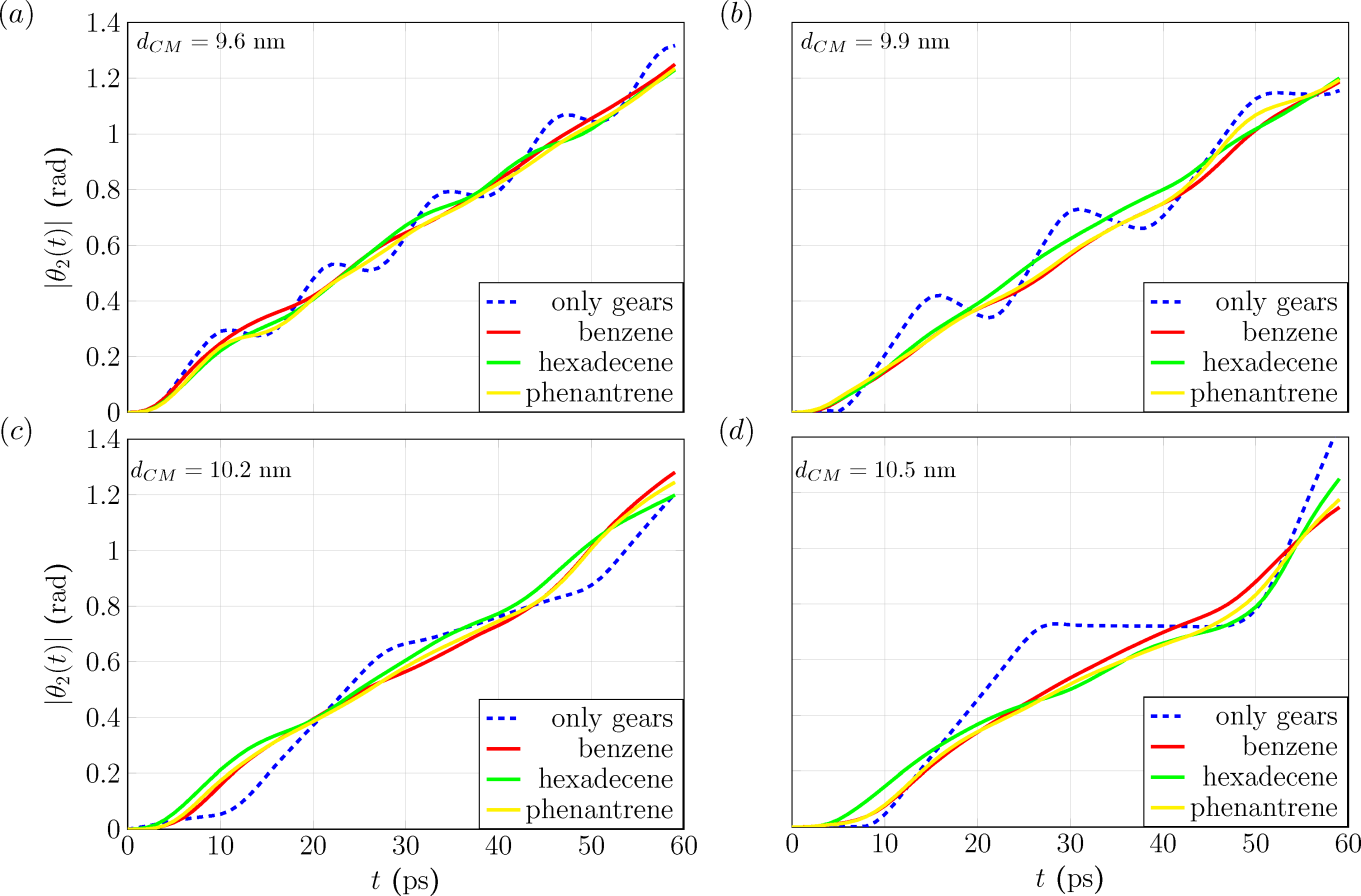
Trajectories of the rotation angle of the second gear θ_2_(*t*) without lubricant (blue dashed lines) and with lubricants, that is, benzene (red), hexadecene (green) and phenanthrene (yellow) from MD simulations within 60 ps for different center-of-mass distances *d*_CM_ = (a) 9.6 nm, (b) 9.9 nm, (c) 10.2 nm and (d) 10.5 nm.

First, we consider the setup in Figure 1 with protocol A to compare how different lubricants can affect rotational transmission. We enforce a constant angular velocity ω = 6.67π rad/ns for the first gear shown on the left in Figure 1. Note that this angular velocity is very high and was chosen in order to make the MD simulation feasible in terms of computation time. In fact, one might expect for that the result from MD simulations might be different for lower speeds. However, we expect that results in the low-speed regime would be qualitatively similar and would be somewhere between our high-speed simulations and quasi-static simulations. However, instead of fixing the angular velocity for atoms in the first gear, we only fix the atoms within the inner cylindrical region, that is, those with radius *r* ≤ 2 nm. In this way, the outer atoms are allowed to deform, which avoids instantaneous torque transfer and makes the simulation more realistic. Then we monitor how the second gear can follow the motion of the first one. The results from the MD simulation within 60 ps for different center-of-mass distances, *d*_CM_ = 9.6, 9.9, 10.2 and 10.5 nm, are shown in Figure 2a,b,c and d, respectively.

The blue dashed lines are trajectories of the rotation angle of the second gear corresponding to the case without lubricants, which exhibit oscillations on top of a linearly increasing trend. One can imagine that when the angular momentum is transferred from the first gear to the second one, the teeth of both gears start jiggling around. Moreover, there is a finite phase-shift or time delay for the second gear compared to the first one, whose trajectory is a linear straight line. This phenomenon is due to the nonzero distance between the teeth of both gears during their collective rotation. This is not expected to happen for ideal, perfectly interlocked gears in contact [43,63–64]. Besides, by increasing *d*_CM_ from 9.6 to 10.5 nm one can see that this time delay behavior becomes more prominent.

For the cases with lubricants, that is, benzene (red), hexadecene (green) and phenantrene (yellow), one can see in Figure 2 that the trajectories are smoother and that oscillation amplitude and time delay for the second gear are reduced. Moreover, this effect is independent of the type of lubricants and of the distance *d*_CM_, although one can still see a small difference between hexadecene (green) and benzene/phenantrene (red/yellow). This is because, unlike benzene and phenantrene, hexadecene has sp^3^ hybridization, which results in a larger monolayer thickness. These important findings imply that lubricants at the nanoscale can be used to synchronize both gears and make the collective rotation closer to the case of rigid bodies. The underlying reason for this behavior is the tendency of the lubricant molecules to fill the gap between gears and to provide a medium for angular momentum transfer at all times, which stabilizes the motion of the second gear. However, more energy is needed to sustain the rotation with the same angular velocity since energy is dissipated into the surrounding lubricant. This has been confirmed by independent constant torque gear rotation simulations in which, for the case with lubricants, the corresponding angular velocity is highly reduced. Note that gear–gear friction or gear–lubricant friction are accounted for within the MD simulation in the form of an irreversible rotational kinetic energy dissipation. The energy of gear rotation can be transferred to deformation energy [49] or to the lubricants due to microscopic Lennard-Jones interactions.

### Angular velocity dependence

**Figure 3:**
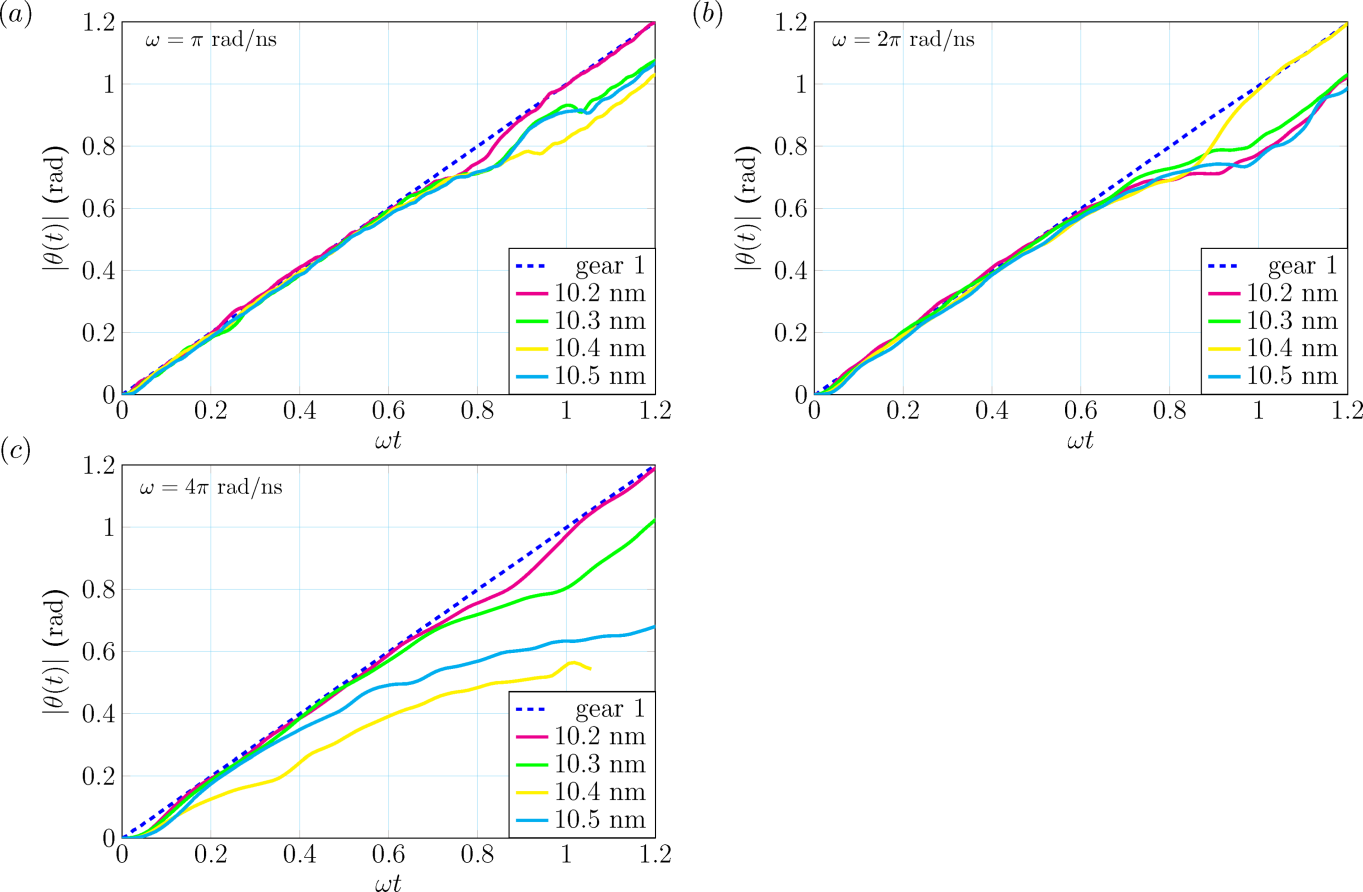
The trajectories from MD simulations of gears lubricated by hexadecene for different angular velocities of the first gear: (a) ω = π rad/ns, (b) ω = 2π rad/ns and (c) ω = 4π rad/ns. The blue dashed lines represent the trajectories of the first gear θ_1_(*t*) and the others are denoting the angle θ_2_(*t*) of the second gear for different center-of-mass distances ranging from 10.2 to 10.5 nm.

From the previous section, we know that the lubricants can assist the transmission of angular momentum between gears. One might still wonder if the angular velocity of the first gear plays any role. Therefore, we performed MD simulations with different initial angular velocities ω = π, 2π and 4π rad/ns (or periods equal to 2000, 1000 and 500 ps). To check if lubricants can protect the surface of the gears, we use protocol B in the MD simulations, which allows for bond formation to happen between gears. Note that the C–C bonds between gears are characterized by two carbon atoms attaching each other within the diamond bond length 1.54 Å [59]). We choose hexadecene for the following discussion. Moreover, we also carried out the simulations with center-of-mass distances *d*_CM_ = 10.2, 10.3, 10.4 and 10.5 nm to investigate how the distance dependency changes with respect to the angular velocity. Since we have different angular velocities, the simulation time is normalized in order to compare the trajectories on equal footing. We plot the trajectories (or angle displacements) θ with respect to the dimensionless time ω*t*. The results are shown in Figure 3. Note that the angle displacements are obtained by integrating the *z*-component of angular velocity vectors over time, where the angular velocity vectors can be calculated by LAMMPS.

The blue dashed lines are denoting trajectories |θ_1_(*t*)| of the first gear and all the other lines represent |θ_2_(*t*)| for different *d*_CM_. One can immediately see that as ω increases, some trajectories of the second gear, especially in the cases of 10.4 and 10.5 nm with ω = 4π rad/ns (yellow and cyan in Figure 3c), have larger phase delays. Note that for the case of 10.4 nm some atoms are lost and hence the simulation was not finished. This is due to the fact that we enforce the first gear to keep rotating after C–C bond formation between gears, which results in the dissociation of atoms from the teeth and the short-range repulsion in the Lennard-Jones contribution. This makes the corresponding atom suddenly move outside the simulation box in very short time and the simulation stops. Note that this is an artifact of the AIREBO potential. If we only use the REBO potential then the bond-breaking process is much more stable (as we found out in independent simulations). Also, there are some interesting phenomena happening in the cases of *d*_CM_ = 10.2 nm with ω = π rad/ns (magenta in Figure 3a), *d*_CM_ = 10.4 nm with ω = 2π rad/ns (yellow in Figure 3b) and *d*_CM_ = 10.2 nm with ω = 4π rad/ns (magenta in Figure 3c) where you can find the trajectories to have some delay around ω*t* = 0.8 and become coherent again at around ω*t* = 1. After monitoring the full trajectories, we found that the time duration between ω*t* = 0.8 and ω*t* = 1 corresponds to the transition from the first step (0 to 60°) to the second step (60 to 120°) of the rotation. The underlying reason for the delays happening at ω*t* = 0.8 can be understood as follows (see also the movie in Supporting Information File Supporting Information File 1): When the first step of rotation is finished, the distance between teeth is too large to effectively transfer angular momentum through the lubricant and this results in a time delay. However, when the other teeth get closer, they will then quickly repel each other again via the lubricant and slightly accelerate the second gear until they become coherent again.

**Figure 4:**
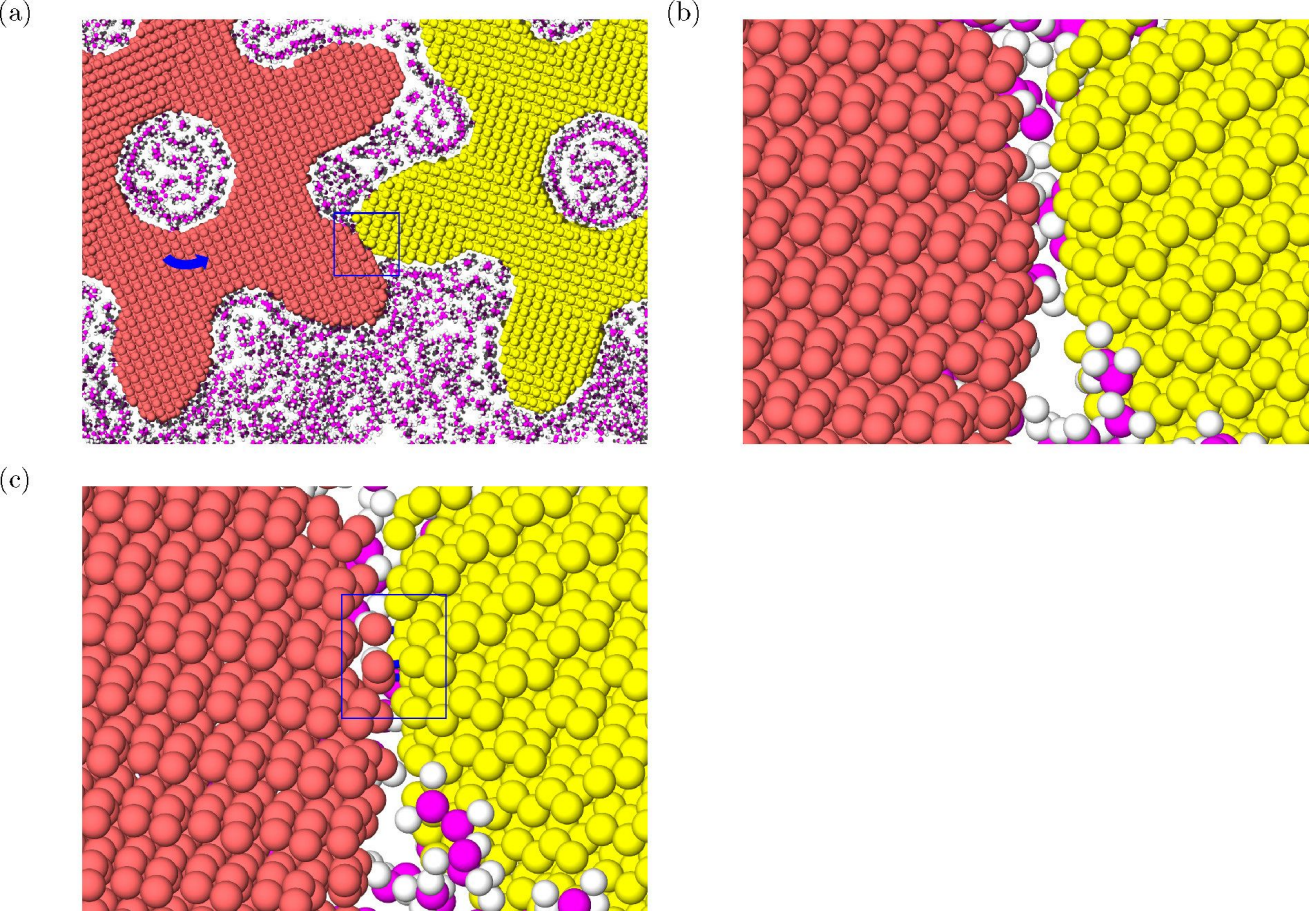
Snapshots for frames around the bond formation event from the MD simulation with center-of-mass distance *d*_CM_ = 10.4 nm and angular velocity ω = π rad/ns. (a) Top view of the frame at *t* = 299 ps (θ_1_ ≈ 0.94 rad or 54°). Zoom-in of the area around the teeth (b) before and (c) after the bond formation event (*t* = 300 ps). The new bonds between gear carbon atoms (red and yellow for left and right, respectively) are marked in blue.

As for the other cases, one can see that a net delay happens. Consider, for instance, ω = π rad/ns with *d*_CM_ = 10.4 nm (yellow in Figure 3a): After investigating the full trajectory (see Figure 4), we find that at the end of the transition from the first step to the second step (around 54°) some lubricant molecules are repelled from the tooth.

This process results in a net phase delay and eventually makes two gears to have direct contact with each other and to form additional C–C bonds between them (shown in Figure 4b,c). For the cases with *d*_CM_ = 10.4 and 10.5 nm, these phenomena happen at the early stage of the rotation. One can see that the phase delay occurs when ω*t* ≈ 0.1 (the first gear rotates only 5°) and ω*t* ≈ 0.4 (around 15°) for the cases of 10.4 and 10.5 nm, respectively. This implies that for larger *d*_CM_ and ω transmission of torque via lubricants is less effective.

### Bond formation between gears

From the previous discussion, we know that the bond formation between gears results in a net phase delay for the second gear. Therefore, we want to investigate when the bond formation happens for different center-of-mass distances and angular velocities of the first gear. The results for different *d*_CM_ and ω are shown in Table 1.

**Table 1:** Analysis of C–C bond formation between gears for different center-of-mass distances *d*_CM_ and angular velocities of the first gear ω in units of nm and rad/ns, respectively. The MD simulations are all performed with normalized simulation time ω*t* = 1.2 corresponding to two steps of rotation of the first gear. The values in the table represent the angle displacement of the first gear when the bond formation happens, the number in parentheses denotes the number of bonds formed within that time frame and a minus symbol (−) denotes no bond formation until the end of the simulation.

	*d*_CM_			
	
ω [rad/ns]	10.2 nm	10.3 nm	10.4 nm	10.5 nm

2π/3	50.4°(1)	52.0°(1)	61.8°(1)	−
4π/5	−	−	−	59.9°(1)
π	−	59.0°(1)	54.2°(1)	59.9°(1)
4π/3	−	54.0°(1)	24.0°(1)	−
2π	50.8°(1)	54.0°(1)	−	54.4°(1)
4π	−	54.0°(1)	28.8°(11)	38.2°(1)

Since the angular velocities are different, we use the angle instead of the time to compare different simulations. The values in the table show the angles for the first gear when C–C bond formation occurs between gears. One can see that, for different *d*_CM_, there is no clear trend for the formation of bonds. Also, we find that most bond formations happen around 50 to 60°, which correspond to the transition from the first step to the second step since teeth are getting closer and lubricant molecules have a higher probability to be squeezed out. As for the angular velocity, we find indeed that higher angular velocities could affect the bond formation. For instance, for ω = 4π/3 rad/ns and *d*_CM_ = 10.4 nm, we have a small angle θ_1_ = 24.0°. Moreover, for ω = 4π rad/ns and *d*_CM_ = 10.4 and 10.5 nm, we have also two small angles θ_1_ = 28.8° and 38.2°, which means the angular velocity is very high and the lubricant molecules cannot follow the motion of the first gear adiabatically and therefore are repelled very quickly.

## Conclusion

In this study, we performed MD simulations for a system of two diamond solid-state gears with different lubricants (benzene, hexadecene and phenanthrene). We found that lubricants can be used to synchronize the collective rotations in both gears by filling the gap for better angular momentum transfer and this effect is independent of the type of the lubricant molecule. Moreover, we found that, as the angular velocity of the first gear increases, the net phase delay between gears becomes prominent and this can be traced to the C–C bond formation between gears. Further investigation of bond formation under different conditions shows that most bond formations happen during the transition from the first step to the second step. Also, the center-of-mass distance does not affect the bond formation while the angular velocity can indeed increase the probability of bond formation since lubricant molecules cannot follow the first gear adiabatically.

Knowing that the lubricants can help synchronize the rotational transmission, future studies will need to address if one can functionalize the gear surfaces with specific chemical groups that can prevent the bond formation but at the same time keep gears being perfectly interlocked. Also, a multiscale simulation involving continuum modelling in combination with MD simulations would be helpful to connect the atomistic description with macroscopic behavior, for example, as given by the Stribeck curve [5]. A substrate can also provide significant friction due to electron or phonon excitations [65], which cannot be captured by a Lennard-Jones plane as used in our simulations. To further investigate those open questions, a more powerful pair potential such as the reactive force field (ReaxFF) [66] or a deep learning force field [67] approach might be suitable to address the problem. Finally, we hope that full atom simulations in combination with further advances in the fabrication and chemical synthesis techniques will improve the design of solid-state gears at the microscopic scale.

## Supporting Information

File 1 Animated GIF showing the rotation steps.
