# Supplementary figures and images for: Effect of lubricants on the rotational transmission between solid-state gears
Source: Beilstein J Nanotechnol. 2022 Jan 5;13:54–62. doi: 10.3762/bjnano.13.3 (PMC8744455; doi:10.3762/bjnano.13.3)

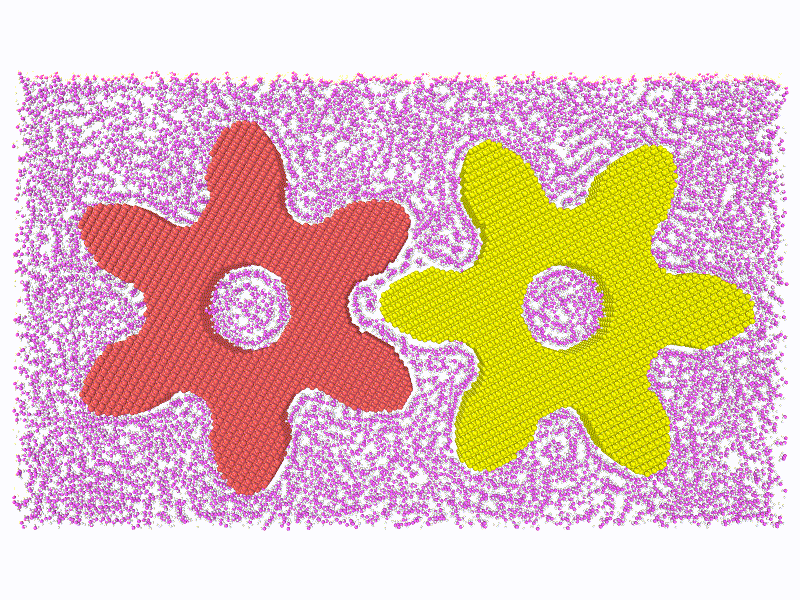

Supplement: File 1 — Animated GIF showing the rotation steps. [file Beilstein_J_Nanotechnol-13-54-s001.gif]
